# Acute phase characteristics and long-term complications of pulmonary embolism in COVID-19 compared to non-COVID-19 cohort: a large single-centre study

**DOI:** 10.1186/s12890-023-02323-9

**Published:** 2023-01-18

**Authors:** A. Franco-Moreno, D. Brown-Lavalle, M. Campos-Arenas, N. Rodríguez-Ramírez, C. Muñoz-Roldán, A. I. Rubio-Aguilera, N. Muñoz-Rivas, J. Bascuñana-Morejón de Girón, E. Fernández-Vidal, E. Palma-Huerta, S. Estévez-Alonso, B. Rodríguez-Gómez, S. Manzano-Valera, R. Pedrero-Tomé, M. Casado-Suela, C. Bibiano-Guillén, M. Mir-Montero, J. Torres-Macho, A. Bustamante-Fermosel, the Infanta Leonor Thrombosis Research Group, E. Moya-Mateo, B. Mestre-Gómez, R. M. Lorente-Ramos, J. Rogado, B. Obispo, D. Salazar-Chiriboga, T. Sáez-Vaquero, A. Abad-Motos, C. Cortina-Camarero, A. Such-Díaz, E. Ruiz-Velasco, F. Sierra-Hidalgo, M. de Carranza-López, M. A. Herrera-Morueco, M. Akasbi-Montalvo, P. Medrano-Izquierdo, E. Mariscal-Gómez, K. Marín-Mori, C. Figueras-González, S. López-Lallave, D. Díaz-Díaz, C. Mauleón-Fernández, J. Martín-Navarro, P. Torres-Rubio, C. Matesanz, M. J. Moro-Álvarez, J. A. Hernández-Rivas

**Affiliations:** 1 Internal Medicine Department, Hospital Universitario Infanta Leonor–Virgen de la Torre, Gran Via del Este Avenue, 80, 28031 Madrid, Spain; 2 Radiology Department, Hospital Universitario Infanta Leonor–Virgen de la Torre, Madrid, Spain; 3 Internal Medicine Department, Hospital Universitario Doce de Octubre, Madrid, Spain; 4 EPINUT-UCM (Ref. 920325) Investigation Group, Universidad Complutense de Madrid, Madrid, Spain; 5 Fundación para la Investigación e Innovación Biomédica de los Hospitales Universitarios Infanta Leonor y del Sureste, Madrid, Spain; 6 Emergency Department, Hospital Universitario Infanta Leonor–Virgen de la Torre, Madrid, Spain

**Keywords:** Case-control study, COVID-19, Pulmonary embolism, Risk stratification, Severity, Long-term complications

## Abstract

**Background:**

To compare the severity of pulmonary embolism (PE) and the long-term complications between patients with and without COVID-19, and to investigate whether the tools for risk stratification of death are valid in this population.

**Methods:**

We retrospectively included hospitalized patients with PE from 1 January 2016 to 31 December 2022. Comparisons for acute episode characteristics, risk stratification of the PE, outcomes, and long-term complications were made between COVID and non-COVID patients.

**Results:**

We analyzed 116 (27.5%) COVID patients and 305 (72.4%) non-COVID patients. In patients with COVID-19, the traditional risk factors for PE were absent, and the incidence of deep vein thrombosis was lower. COVID patients showed significantly higher lymphocyte count, lactate dehydrogenase, lactic acid, and D-dimer levels. COVID patients had PE of smaller size (12.3% vs. 25.5% main pulmonary artery, 29.8% vs. 37.1% lobar, 44.7% vs. 29.5% segmental and 13.2% vs. 7.9% subsegmental, respectively; *p* < 0.001), less right ventricular dysfunction (7.7% vs. 17.7%; *p* = 0.007) and higher sPESI score (1.66 vs. 1.11; *p* < 0.001). The need for mechanical ventilation was significantly higher in COVID patients (8.6% vs. 1.3%; *p* < 0.001); However, the in-hospital death was less (5.2% vs. 10.8%; *p* = 0.074). The incidence of long-term complications was lower in COVID cohort (*p* < 0.001). PE severity assessed by high sPESI and intermediate and high-risk categories were independently associated with in-hospital mortality in COVID patients.

**Conclusion:**

The risk of in-hospital mortality and the incidence of long-term complications were lower in COVID-19. The usual tools for risk stratification of PE are valid in COVID patients.

## Background

Pulmonary embolism (PE) is a known complication of SARS-CoV-2 infection (COVID-19), implying a worse prognosis [1–4]. COVID-19 leads to a hyperinflammatory state that generates an abnormal coagulation system activation and direct endothelial injury resulting in thrombosis [5, 6]. Increased neutrophilic infiltration of lung capillaries and thrombosis in the small vessels in autopsy series of patients with COVID-19 have been reported, despite prophylactic anticoagulation [7–9].

PE characteristics in COVID patients seem to differ compared to non-COVID patients. Lung thrombosis in COVID-19 is frequently found in peripheral arteries [10], and the reported incidence of concomitant deep vein thrombosis (DVT) is lower [11]. These data suggest that in situ vascular thrombosis instead of distal embolism is this population’s underlying physiological mechanism of PE.

The potential impact of PE on the prognosis of COVID patients has not been appropriately assessed. The European Society of Cardiology (ESC) has proposed a risk stratification model for death in patients with acute PE based on clinical scores (simplified Pulmonary Embolism Severity Index [sPESI]), right ventricle dysfunction (RVD) and elevated serum troponin [12]. The ability of this model to predict early mortality (in-hospital or 30 days after PE diagnosis) in COVID-19 patients has not been previously described. On the other hand, the frequency of RVD, the prognostic accuracy of the sPESI score, and the correlation between these indicators of the severity of PE and patient outcomes have seldom been reported. Finally, PE complications during follow-up such as chronic thromboembolic pulmonary hypertension (CTEPH) and recurrence of venous thromboembolism (VTE) remain to be established.

Bearing in mind all these gaps in the current knowledge, the present study aimed to describe the clinical, analytical, and radiological characteristics, the correlation between severity assessment and outcomes of PE during the acute phase, and the long-term complications, including recurrence of VTE and CTEPH in COVID patients, and to compare them with a non-COVID cohort.

## Methods

### Study design and setting

This retrospective case-control study enrolled adult patients diagnosed with PE attending the emergency department and medical wards at Hospital Universitario Infanta Leonor–Virgen de la Torre. Patients who underwent a computed tomography pulmonary angiogram (CTPA) for suspected PE were located through the computerized registry of the Radiology department. COVID patients with PE diagnoses between 1 January 2020 and 31 December 2021 formed the cases. The diagnosis of COVID-19 was established on SARS-CoV-2 RNA detection in a nasopharyngeal swab by reverse transcriptase polymerase chain reaction (RT-PCR) or antigen detection test. The indication for CTPA was based on medical criteria. The main reasons for suspecting PE were development or worsening dyspnoea, desaturation, or significantly elevated D-dimer levels. The control group was made up of non-COVID patients admitted to the hospital with a diagnosis of PE between 1 January 2016 and 31 December 2019, just four years before the COVID-19 pandemic. A Control group was formed, selecting three non-COVID patients for every COVID case (case/control ratio of 1:3).

Variables related to the acute episode of PE were collected from electronic medical records using a standardized form: Baseline characteristics, traditional risk factors for PE, CTPA findings (thrombi location [main pulmonary arteries, lobar arteries, segmental or subsegmental arteries], number of lungs affected by PE [uni– or bilateral], presence of pulmonary infarction and pleural effusion), hemodynamic data, cardiac troponin levels, the presence of RVD, concomitant DVT and the sPESI score. We calculated the risk stratification for death using the classification of the ESC [12]. Patients’ risk was classified as high (shock or hypotension < 90 mmHg), intermediate-high (RVD and elevated troponin), intermediate-low (RVD or increased troponin or none), and low (sPESI 0). Variables related to the long-term complications of PE during follow-up, including CTEPH and recurrence VTE, were also recorded.

The primary outcome in the present study was the combination of in-hospital all-cause mortality and the need for mechanical ventilation. We specifically investigated the causes of death. We analyzed the ability of the risk stratification model for death in PE developed in the general population for predicting mortality in COVID patients. Second, we analyzed the severity of PE according to PE size (which was classified by the localization of the most proximal artery involved), the presence of RVD in the transthoracic echocardiography, and the sPESI score. Finally, we also investigated the recurrence of VTE and CTEPH. At the time of the present analysis, all patients had finished the index episode (they had been discharged home or died during hospital admission). After discharge, patients regularly were attended follow-up appointments in professional consultation of VTE for at least 6 months.

### Pulmonary artery computed tomography protocol

In order to detect pulmonary emboli, a CTPA was performed after intravenous administration of contrast material (Iohexol 300 mg I/ml) to achieve an opacification of the pulmonary arterial tree and demonstrate intravascular filling defects.

The patient was positioned supine with his arms above his head, and an initial scout was made from the apical zone to the diaphragmatic cupola.

We use the bolus-tracking technique: before contrast administration, a slice below the carina of the trachea is used to locate the pulmonary trunk, and a region of interest (ROI) is placed over the center of the vessel.

Attenuation values in this ROI are registered in the unenhanced image and then after administration of contrast media every 0.7 s until a pre-established threshold is met (in our center is 80 HU) and scanning starts in a craniocaudal direction and complete inspiration. A total volume of contrast of 50–60 ml is used with an injection rate of 4 ml/s. Images are reconstructed with a 1 mm section thickness and an interval of reconstruction of 1 mm.

### Statistical analysis

Qualitative variables were expressed as absolute and relative frequency distributions, while quantitative variables were expressed as median with interquartile range (IQR). Differences between case and control groups were assessed by the Chi2 test or Fisher exact test for qualitative variables and the Student’s t-test or the non-parametric Mann–Whitney U-test for quantitative variables according to whether or not they conformed to a normal distribution, respectively. Adjusted odds ratios (OR) with a 95% confidence interval (CI) were calculated in a multivariable analysis. A *p* < 0.05 was considered statistically significant. All statistical analyses were performed using SPSS software, version 25.0 (IBM Corp, Armonk, NY, USA).

### Ethics

We conducted a single-center observational analytical study based on a retrospective cohort, following the STROBE recommendations for observational studies [13]. The study was approved by the Ethics Committee (CEI) of the Hospital Universitario Clínico San Carlos (code 22/145-E). It was carried out following the Declaration of Helsinki and Good Clinical Practice guidelines. Informed consent was waived because of the study’s retrospective nature, and clinical data were anonymized.

## Results

**Fig. 1:**
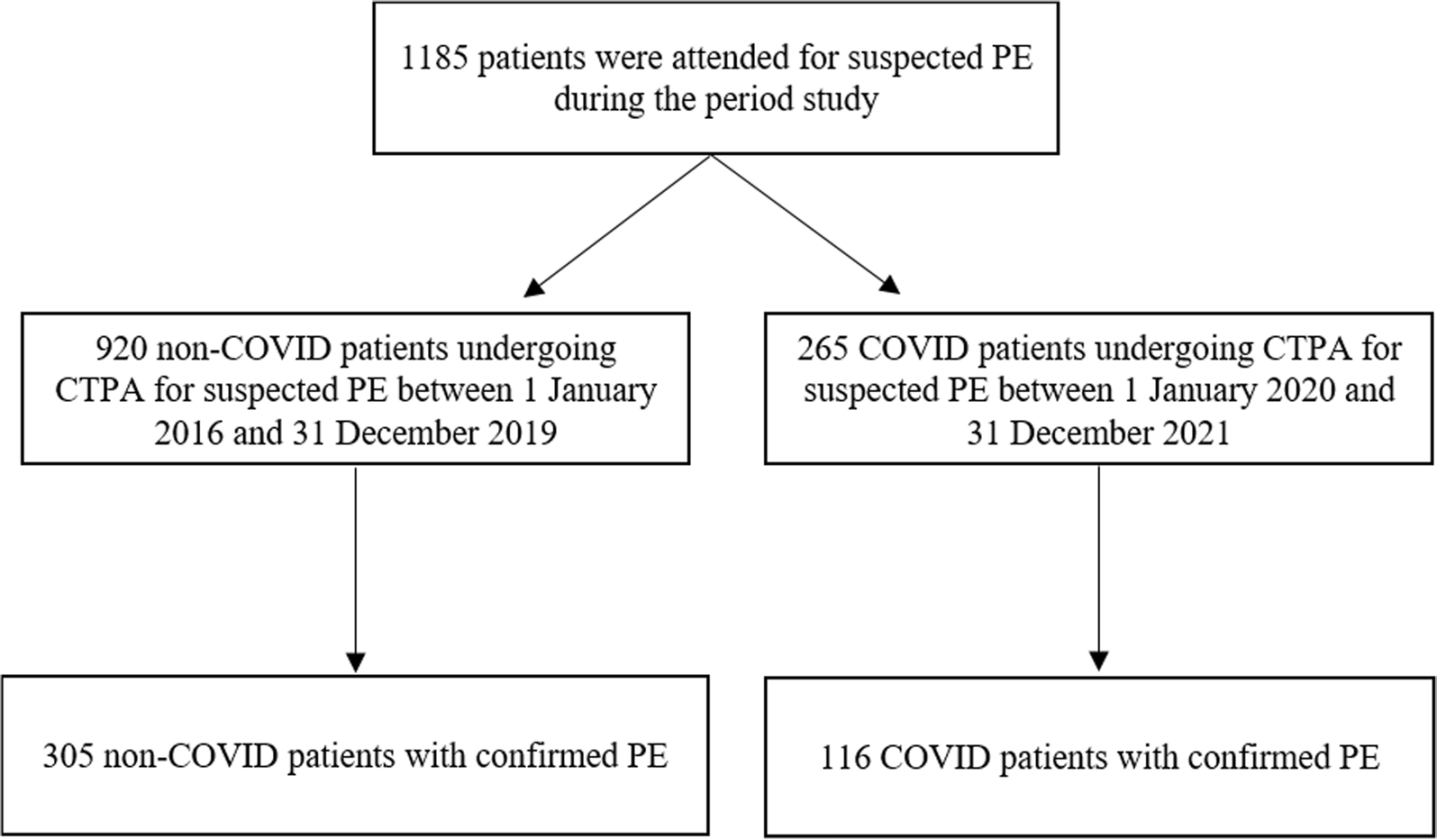
Study flow chart. *CTPA* computed tomography pulmonary angiogram, *PE* pulmonary embolism

**Fig. 2:**
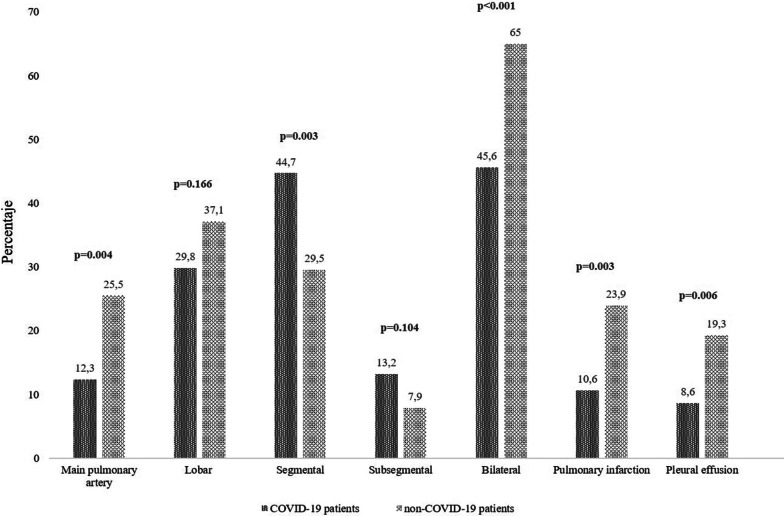
Imaging findings on computed tomography pulmonary angiogram in patients with pulmonary embolism, comparing COVID and non-COVID patients

**Fig. 3:**
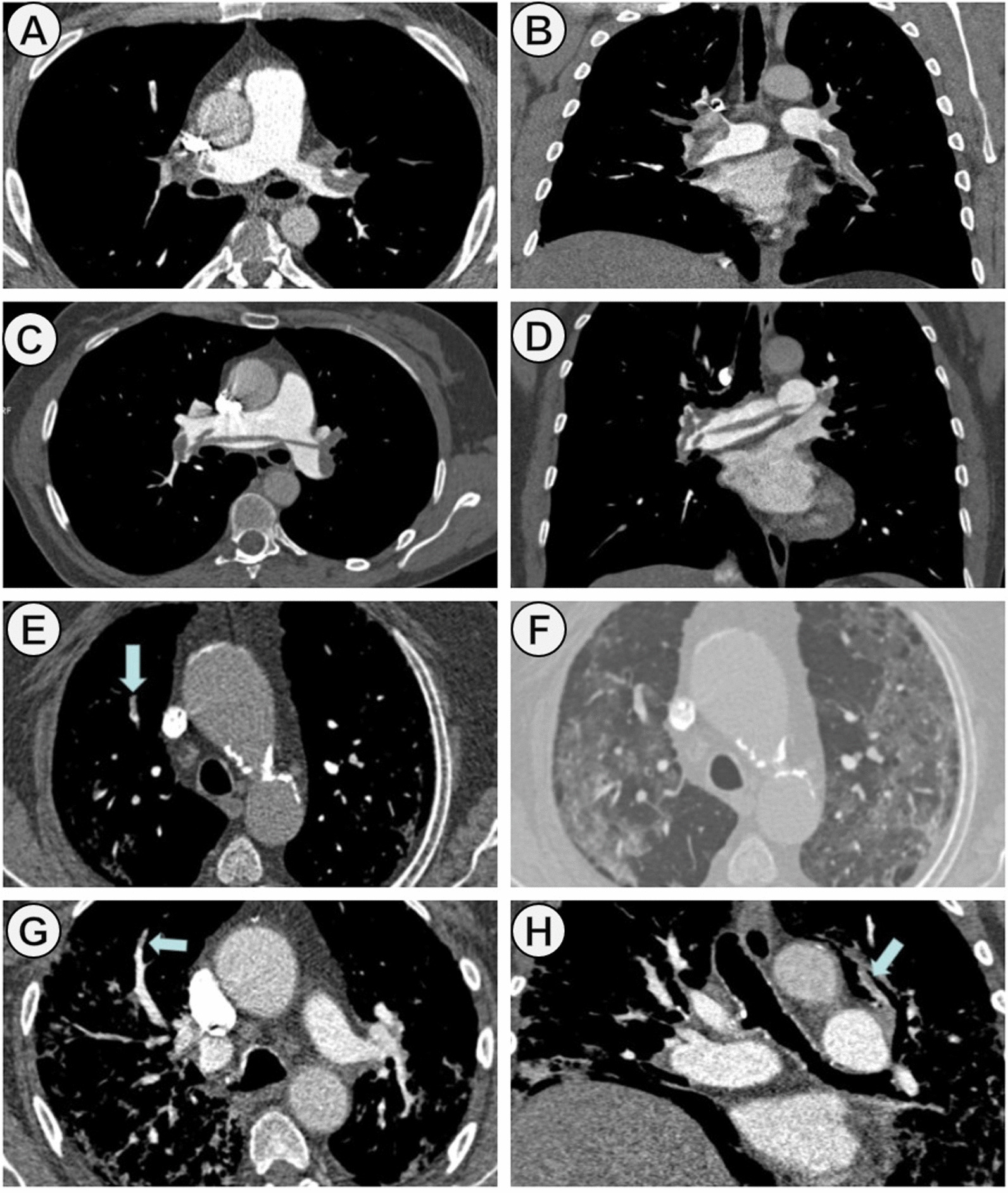
PE images on CTPA in COVID and non-COVID patients: **A**–**D** correspond to axial and coronal images in two non-COVID patients where filling defects within the pulmonary vasculature are central (**A** and **B** in the right and left pulmonary arteries; **C** and **D** in the main trunk of the pulmonary artery (“saddle pulmonary embolism”); **E**–**H** represent peripheral pulmonary embolism in two COVID patients. Thrombi are seen in segmental arteries in right upper lobe in (**E**)–(**F**) and in segmental arteries of both lungs. In these two patients there were also parenchymal abnormalities (ground glass opacities and septal thickening) better seen in CT lung window (**F**). *PE* pulmonary embolism, *CTPA* computed tomography pulmonary angiogram

A total of 421 patients with PE were included during study period. Of them, 116 (27.5%) COVID patients and 305 (72.4%) non-COVID patients (Fig. 1). Baseline characteristics of case and control cohorts are shown in Table 1. The median age was 67.2 (± 14.4) years in COVID patients and 66.2 (± 17.18) in non-COVID patients. 57 patients (49.1%) and 133 patients (43.6%) were male in the case and control groups, respectively. The common comorbidities were hypertension (52.9%), dyslipidemia (32.0%), and diabetes mellitus (18.5%). From 1st January to 31th December 2021, 6.0% (7/116) of COVID-19 patients had received at least one COVID-19 vaccine dose. Compared to non-COVID patients, in COVID cohort, classic risk factors for PE were less frequent, and they had a lower incidence of concomitant DVT. Tachycardia and tachypnea at the time of PE diagnosis were most found in COVID patients. However, the hemodynamic instability (systolic blood pressure < 90 mmHg) was significantly lower (*p* < 0.001). Among laboratory parameters, there were statistically significant differences in lymphocytes, lactate dehydrogenase (LDH), lactic acid, and D-dimer levels. The main pulmonary and lobar arteries were more frequently involved in non-COVID patients, while thrombi were more frequently restricted to segmental and subsegmental arteries in COVID patients (Fig. 2). Remarkably, the presence of bilateral lung affection, pleural effusion and pulmonary infarction was more frequent in non-COVID patients (*p* < 0.001) (Fig. 3). The presence of RVD on transthoracic echocardiography was less frequent in COVID patients compared to the control group (7.9% vs. 17.7%, respectively; *p* = 0.012). Regarding therapies for COVID-19, 76.7% (89/116) patients received corticosteroids, 44.8% (52/116) anti-cytokine antibodies, and 39.6% (46/116) antiviral drugs. COVID patients had a higher sPESI score than non-COVID patients (*p* < 0.001; Table 2). Statistically significant differences in the need for mechanical ventilation between case and control groups were observed (8.6% vs. 1.3%; *p* < 0.001). Thirty-nine patients died during hospitalization (9.26%). The risk of in-hospital death was higher in non-COVID patients, although no statistically significant differences were observed (10.8% vs. 5.2%; *p* = 0.074). In COVID cohort, five patients (83.3%) died due to severe acute respiratory distress syndrome and one patient due to sepsis. In non-COVID patients, the causes of death were active cancer (63.6%), fatal PE (24.2%), sepsis (9.0%), and one patient who died due to esophageal variceal bleeding. In non-cancer patients subgroup, mortality was 4.5% (5/111) in COVID-19 patients and 2.8% (6/211) in non-COVID-19 cohort. No low sPESI patients in the two groups died (Table 2). In-hospital mortality was higher in patients with central o lobar thrombi location in the case and control group (50% [3/6] and 57.7% [19/33], respectively; *p* < 0.001); However, this correlation was not observed in patients with RVD. The categorisation of the PE risk was calculated in 48 COVID patients and 194 non-COVID patients in whom data on the sPESI score, RVD and serum troponin were available (Table 3). Four and 11 patients died in COVID and non-COVID cohort, respectively. Of them, all COVID patients were classified into the intermediate-high-risk category, and 10 non-COVID patients (90.9%) were classified into the intermediate-high and high-risk categories.

**Table 1:** Characteristics of patients with pulmonary embolism in acute phase stratified by the presence or absence of COVID-19.

Variables	COVID patients (n = 116)	Non-COVID patients (n = 305)		*p* value
Demographic characteristics				
Age, years (media [SD])	67.21 (± 14.49)	66.28 (± 17.18)		0.987
Male, n (%)	57 (49.1)	133 (43.6)		0.308
Setting				
Emergency department	67 (57.8)	189 (62.0)		0.429
Medical wards	49 (42.2)	116 (38.0)		0.429
Classic risk factors for PE, n (%)				
Previous venous thromboembolic event	2 (1.7)	31 (10.2)		< 0.001
Obesity (BMI > 30 kg/m^2^)	49 (42.2)	103 (33.8)		0.111
Recent immobilization (last month^†^)	5 (4.3)	51 (16.8)		< 0.001
Recent surgery (last month)	3 (2.6)	31 (10.1)		0.011
Thrombophilia	1 (0.9)	21 (6.9)		0.013
On oestrogen therapy	7 (6.0)	48 (15.7)		0.008
Active cancer	15 (12.9)	94 (30.8)		< 0.001
Cardiovascular risk factors, n (%)				
Diabetes	25 (21.6)	53 (17.4)		0.325
Hypertension	66 (56.9)	157 (51.5)		0.319
Dyslipidemia	44 (37.9)	91 (29.8)		0.112
Smoking	12 (10.3)	21 (6.8)		0.011
Other comorbidities, n (%)				
Asthma	13 (11.2)	19 (6.2)		0.085
COPD	7 (6.0)	32 (10.4)		0.159
Cerebrovascular disease	10 (8.6)	13 (4.3)		0.079
Coronary artery disease	6 (5.2)	14 (4.6)		0.802
Chronic kidney disease	5 (4.3)	23 (7.5)		0.235
Chronic heart failure	5 (4.3)	16 (5.2)		0.694
Chronic liver disease	5 (4.3)	3 (1.0)		0.026
Autoimmune disease	11 (9.5)	13 (4.3)		0.039
Vital signs at the time of PE the diagnosis				
Heart rate, bpm (media [SD])	95 (± 14)	73 (± 13)		0.047
Respiratory rate, bpm (media [SD])	27 (± 31)	20 (± 24)		< 0.001
Systolic blood pressure, mmHg (media [SD])	126 (± 21)	127 (± 22)		0.855
Systolic blood pressure < 90 mmHg, n (%)	2 (1.7)	12 (3.9)		< 0.001
O_2_ saturation (media [SD])	90 (± 4)	94 (± 4)		< 0.001
PaO_2_/FiO_2_ ratio (median [IQR])	240 (115–345)	319 (268–395)		< 0.001
Laboratory findings at the time of PE diagnosis				
Leukocytes, cells/mL (median [IQR])	8865 (6954–12,067)	9320 (7580–12,150)		0.225
Neutrophils, cells/mL (median [IQR])	6750 (4725–9725)	6850 (4795–9200)		0.844
Lymphocytes, cells/mL (median [IQR])	1100 (800–1700)	1500 (1000–2100)		< 0.001
Platelets, cells/mL (median [IQR])	240.000 (174.000– 305.000)	235.000 (181.000–290.000)		0.848
Haemoglobin, g/dL (median [IQR])	13.15 (12.10–14.48)	13.50 (12.13–14.90)		0.415
D-dimer, ng/mL [median [IQR])	5530 (2720–15,950)	4200 (2380–9230)		0.036
C-reactive protein, mg/L (median [IQR])	54.50 (11.70–125.60)	47.10 (12.65–95.00)		0.387
Creatinine, mg/dL (median [IQR])	0.88 (0.74–1.13)	0.92 (0.80–1.14)		0.270
Lactate dehydrogenase, UI/L (median [IQR])	265 (213–352)	222 (184–288)		< 0.001
Lactic acid, mmol/L (median [IQR])	2.46 (1.87–3.21)	1.75 (1.14–2.40)		< 0.001
Troponin, ng/L (median [IQR])	0.43 (0.01–0.85)	0.12 (0.01–0.60)		0.068
NT-proBNP, pg/mL (median [IQR])	318 (169–1221)	566 (149–3040)		0.392
Right ventricular dysfunction*	9 (7.7)	54 (17.7)		0.007
Deep vein thrombosis, n (%)**	14 (12.0)	100 (32.7)		< 0.001
Outcomes				
Transfer to ICU, n (%)	11 (9.5)	15 (4.9)		0.084
Need for mechanical ventilation, n (%)	10 (8.6)	4 (1.3)		< 0.001
In-hospital death, n (%)	6 (5.2)	33 (10.8)		0.074
Bleeding in acute phase	4 (3.4)	23 (7.6)		0.124
Length of stay, days (median [IQR])	9.5 (6.0–20.2)	4.5 (1.0–12.2)		< 0.001
Long-term complications				
Recurrence VTE, n (%)	0	Provoked PE 12 (3.9)	Unprovoked PE 25 (8.1)	< 0.001
CTEPH, n (%)	9 (7.7)	34 (11.1)		0.215

*SD* Standard deviation, *PE* pulmonary embolism, *BMI* body mass index, *COPD* chronic obstructive pulmonary disease, *IQR* interquartile range, *PaO*_*2*_*/FiO*_*2*_ partial pressure of arterial oxygen/fraction of inspired oxygen (FiO_2_), *ICU* intensive care unit, *sPESI* simplified pulmonary embolism severity index, *VTE* venous thromboembolism, *CTEPH* chronic thromboembolic pulmonary hypertension

*Missing values: 55.1% in COVID patients and 14.0% in non-COVID cohort

**Missing values: 19.8% in COVID patients and 13.7% in non-COVID cohort

^†^Totally confined to bed or chair > 3 days

**Table 2:** Comparison of sPESI score between COVID and non-COVID patients and the correlation with the in-hospital mortality

	COVID patients	Non-COVID patients	*p* value
sPESI (media [SD])	1.66 (± 1.14)	1.11 (± 1.04)	< 0.001
Points, n (%)			
0 points	24 (20.68)	100 (32.78)	0.015
1 point	24 (20.68)	111 (36.39)	0.002
2 points	41 (35.34)	65 (21.31)	0.003
3 points	21 (18.10)	18 (5.90)	< 0.001
≥ 4 points	6 (5.17)	11 (3.60)	0.466
Risk stratification, n (%)			
High risk (≥ 1 point)	92 (79.31)	205 (67.21)	0.015
Low risk (< 1 point)	24 (20.68)	100 (32.78)	0.015
In-hospital mortality, n (%)			
High risk	6 (100)	33 (100)	0.024
Low risk	0	0	‒

*sPESI* simplified pulmonary embolism severity index, *SD* standard deviation

**Table 3:** Risk stratification of the pulmonary embolism in COVID and non-COVID patients and the correlation with the in-hospital mortality

Risk stratification	COVID patients (n = 48)	Non-COVID patients (n = 194)	*p* value
High, n (%)	0	9 (4.7)	< 0.001
Intermediate, n (%)			
Intermediate-high	5 (10.4)	24 (12.3)	0.578
Intermediate-low	35 (72.9)	115 (59.2)	0.021
Low, n (%)	8 (16.7)	46 (23.8)	< 0.001
In-hospital mortality, n (%)			
High risk	0	3 (27.2)	< 0.001
Intermediate risk			
Intermediate-high	4 (100)	7 (63.7)	0.089
Intermediate-low	0	1 (9.1)	0.056
Low risk	0	0	–

During the first 6 months after stopping anticoagulant therapy, there were no recurrent VTE in COVID patients and 37 (12.1%) in non-COVID cohort (12 patients with provoked PE by a transient risk factor and 25 patients with unprovoked PE). The incidence of CTEPH was 7.7% (9/116) in COVID patients and 11.1% (34/305) in the control group, after a median follow-up of 6 months.

## Discussion

In this retrospective analysis of patients diagnosed with PE in the emergency department and medical wards, PE characteristics differ between COVID and non-COVID patients. From a clinical point of view, in patients with COVID-19, the traditional risk factors for PE were absent. Moreover, concerning the analytical data, markers of systemic inflammation were more frequent. Finally, from the radiological point of view, patients with COVID-19 showed a more distal pattern of thrombi location on the CTPA and a lower incidence of DVT. These results is in line with a recent analysis of the SIESTA group cohort [14]. In keeping with this, a meta-analysis showed that segmental and sub-segmental pulmonary arteries were more frequently involved compared to main and lobar arteries [15]. In contrast with classic PE physiopathology, all these findings suggest that thrombus in SARS-CoV2 infection could frequently develop “in situ” in the lungs, favored by the hyperinflammatory state and the direct pulmonary endothelial injury that SARS-CoV-2 infection generates [13].

On 27 December 2020, Spain started to administer vaccination against COVID-19. In most COVID-19 patients, PE was diagnosed in 2020. Therefore, the vaccination rate was low.

Our results show that although severity markers, including sPESI score, the need for mechanical ventilation, or a longer length of stay, were more frequent in COVID patients, in-hospital mortality was lower. These results disagree with the previous analysis of the SIESTA and PEPCOV research teams [16]. In this study conducted by Miró et al. in 62 Spanish and 16 French hospitals, the in-hospital mortality was higher in COVID patients. We found two potential explanations for our finding. First, the higher prevalence of advanced-stage cancers as a cause of death in non-COVID-19 patients. In fact, in non-cancer patients subgroup, in-hospital mortality was higher in COVID-19 when comparing non-COVID cohort. In addition, the central thrombi location conditions a worse prognosis [17]. Interestingly, the RVD had not prognostic value. Nonetheless, caution is recommended when interpreting our data. It is difficult to isolate the adverse effects of death in COVID-19 (essentially lung involvement due to acute respiratory distress syndrome) from the adverse effects of PE. Therefore, the essential role of PE in mortality in COVID infection remains to be elucidated.

Our study shows in COVID patients what was described in non-COVID patients: higher sPESI scores and the intermediate and high-risk category of PE are associated with higher mortality risk. Accordingly, these findings suggest that the usual tools for PE risk stratification may be valid even in COVID patients [12, 18].

Finally, to the best of our knowledge, the incidence of long-term complications of PE in COVID-19 has not been previously described. The rate of thrombosis recurrence in patients after a first episode of provoked VTE by a transient risk factor that completes at least three months of anticoagulant treatment is 3.3% per patient-year [19]. Because the risk of recurrence of VTE in these patients is low, it is justified to stop the anticoagulant treatment after 3 months [20]. Our study confirms that the risk of recurrent VTE in COVID infection is low (0% in our series), congruent with the concept of COVID infection as a transitory thrombotic risk factor. The VTE recurrence in COVID was even lower compared to non-COVID patients with provoked PE after stopping the anticoagulant treatment. Therefore, stopping the anticoagulant therapy after three months in these patients seems appropriate. On the other hand, the incidence of CTEPH was lower compared to non-COVID patients due to the distal topography of the PE in COVID-19 infection.

### Limitations

This study has some limitations. First, data collection was retrospective. This limitation, inherent to retrospective chart reviews, may be limited because we mainly analyzed data reliably reported in medical charts, such as CTPA reports with characteristics of thrombi, RVD, outcomes, and recurrence of VTE. However, some items in the sPESI score may not have been adequately reported in the medical notes. Moreover, the present results do not apply to PE diagnosed in critically COVID patients. COVID-19 ICU patients have significant differences compared to emergency and medial wards patients. First, the incidence of PE is higher on UCI patients, and clinical and analytical characteristics are non-comparable. Additionally, sPESI scale predicts in-hospital mortality, and has been validated for the identification of low-risk patients. Therefore, the score does not apply to critical patients with PE [21, 22]. Finally, our study has been performed throughout different COVID-19 waves, including different SARS-CoV-2 variants, and the vaccinated population.

## Conclusion

In conclusion, in this retrospective analysis, PE in patients with COVID-19 showed significant differences compared to non-COVID patients, which supports the role of in situ pulmonary thrombosis. In COVID patients, the risk of in-hospital mortality and the incidence of long-term complications were lower. This is because COVID-19 itself rather than PE conditions the clinical evolution of patients with both diseases. The usual tools for risk stratification of PE, specifically the sPESI and risk stratification model for early death are valid inCOVID-19-associated PE.

## Data Availability

The datasets used and/or analysed during the current study are available from the corresponding author on reasonable request.

## Abbreviations

COVID-19: Coronavirus disease 2019
CTEPH: Chronic thromboembolic pulmonary hypertension
CTPA: Computed tomography pulmonary angiogram
DVT: Deep vein thrombosis
ESC: European Society of Cardiology
PE: Pulmonary embolism
RVD: Right ventricle dysfunction
sPESI: Simplified pulmonary embolism severity index
VTE: Venous thromboembolism
